# Protective but Not Anticonvulsant Effects of Ghrelin and JMV-1843 in the Pilocarpine Model of *Status epilepticus*

**DOI:** 10.1371/journal.pone.0072716

**Published:** 2013-08-28

**Authors:** Chiara Lucchi, Giulia Curia, Jonathan Vinet, Fabio Gualtieri, Elena Bresciani, Vittorio Locatelli, Antonio Torsello, Giuseppe Biagini

**Affiliations:** 1 Department of Biomedical, Metabolic and Neural Sciences, University of Modena and Reggio Emilia, Modena, Italy; 2 Department of Health Sciences, University of Milano-Bicocca, Monza, Italy; 3 Department of Neurosciences, NOCSAE Hospital, Modena, Italy; Beijing Institute of Radiation Medicine, China

## Abstract

In models of *status epilepticus* ghrelin displays neuroprotective effects mediated by the growth hormone secretagogue-receptor 1a (GHS-R_1a_). This activity may be explained by anticonvulsant properties that, however, are controversial. We further investigated neuroprotection and the effects on seizures by comparing ghrelin with a more effective GHS-R_1a_ agonist, JMV-1843. Rats were treated either with ghrelin, JMV-1843 or saline 10 min before pilocarpine, which was used to induce *status epilepticus*. *Status epilepticus*, developed in all rats, was attenuated by diazepam. No differences were observed among the various groups in the characteristics of pilocarpine-induced seizures. In saline group the area of lesion, characterized by lack of glial fibrillary acidic protein immunoreactivity, was of 0.45±0.07 mm^2^ in the hippocampal stratum lacunosum-moleculare, and was accompanied by upregulation of laminin immunostaining, and by increased endothelin-1 expression. Both ghrelin (*P*<0.05) and JMV-1843 (*P*<0.01) were able to reduce the area of loss in glial fibrillary acidic protein immunostaining. In addition, JMV-1843 counteracted (*P*<0.05) the changes in laminin and endothelin-1 expression, both increased in ghrelin-treated rats. JMV-1843 was able to ameliorate neuronal survival in the hilus of dentate gyrus and medial entorhinal cortex layer III (*P*<0.05 vs saline and ghrelin groups). These results demonstrate diverse protective effects of growth hormone secretagogues in rats exposed to *status epilepticus*.

## Introduction

The gastrointestinal hormone ghrelin is synthesized by X/A-like cells in rodents and P/D1 cells in humans and processed for release in response to fasting, mainly by the stomach [1]–[3]. Ghrelin effectively stimulates food intake and growth hormone (GH) release by acting on the GH secretagogue-receptor 1a (GHS-R_1a_) [4]. Numerous ligands for the GHS-R_1a_ have been developed to mimic ghrelin effects [5]–[7]. Among them, hexarelin and JMV-1843, preclinically tested for pituitary disorders [8]–[12], were shown to be potent releasers of GH with better pharmacokinetics than ghrelin. In particular, JMV-1843 appears to be the GHS-R_1a_ ligand with the longest half-life [11]–[13].

Ghrelin and its synthetic analogs exert several additional activities. For example, ghrelin seems to be a key player in the relationship existing between stress, mood disorders and obesity [14]. Moreover, positive effects on the cardiovascular system [15]–[18] and neuroprotection [19] have been described. Indeed, ghrelin administration is able to partially prevent apoptosis in models of transient forebrain ischemia, in which a reduction of the lesion extent has been consistently demonstrated [20]–[22]. Ghrelin was also found to be protective in other models of brain lesion, including kainate [23] and pilocarpine [24] models of *status epilepticus* (SE). Although reports on ghrelin neuroprotective effects are consistent, it is reported that ghrelin can exert some anticonvulsant effects in pentylenetetrazole-treated rats, in the penicillin model of cortical ictogenesis and also in models of chemoconvulsive SE, such as in the case of pilocarpine or kainate treatment (reviewed in ref. [13]). In particular, in the kainate model of SE, ghrelin decreased the severity and duration (60 min instead of 90 min) of convulsions. Thus, it has to be clarified whether neuroprotection is the consequence or not of the ability of ghrelin to diminish the seizure activity induced by chemoconvulsants. On the other hand, lines of evidence suggest that ghrelin is unable to prevent SE induced by pilocarpine [25], [26] and is anyway neuroprotective [24].

Cerebral lesions that follow pilocarpine-induced SE are widespread [27] and depend on SE duration [28]–[31]. No lesion is detectable in animals that do not experience SE [27], whereas even short periods of continuous convulsive activity are accompanied by damage in the hippocampus, amygdala, thalamus, substantia nigra and cerebral cortex [31]. In particular, the medial entorhinal cortex layer III and the hilus of dentate gyrus appear to be the most consistently injured regions [27], [32]. Recently, we found that the stratum lacunosum-moleculare of the CA3 hippocampal region is predictably damaged just after few minutes of exposure to pilocarpine-induced SE [30]–[31]. This lesion presents several characteristics resembling a focal ischemic injury, i.e. complete disappearance of glial fibrillary acidic protein (GFAP)-positive astrocytes and increased perivascular laminin immunostaining [30]–[31]. Similar changes in GFAP and laminin distribution were also found in areas of focal ischemia induced by endothelin-1 (ET-1) [31], a vasoconstrictor peptide upregulated by pilocarpine before the appearance of vasogenic edema [33]. Interestingly, ghrelin was shown to counteract the effects of ET-1 on blood vessels [17], [34]–[35] and this property could represent an additional mechanism participating in the protection of brain regions affected by vascular injuries.

Here, we evaluated whether ghrelin and another GHS-R_1a_ agonist, JMV-1843, could counteract the insurgence of the ischemic-like lesion that we identified in the CA3 region of pilocarpine-treated rats [30]. We have recently tested various GHS-R_1a_ ligands in the pilocarpine model [25], showing that ghrelin and JMV-1843 are unable to inhibit SE induction, so that any possible neuroprotective effect related to these agents could not be due to the prevention of pilocarpine effects. To make a comparison with a previous report on neuroprotection provided by ghrelin [24], we also characterized neuronal cell damage in regions of the hippocampal formation other than CA3.

## Methods

### Animals and treatments

**Figure 1:**
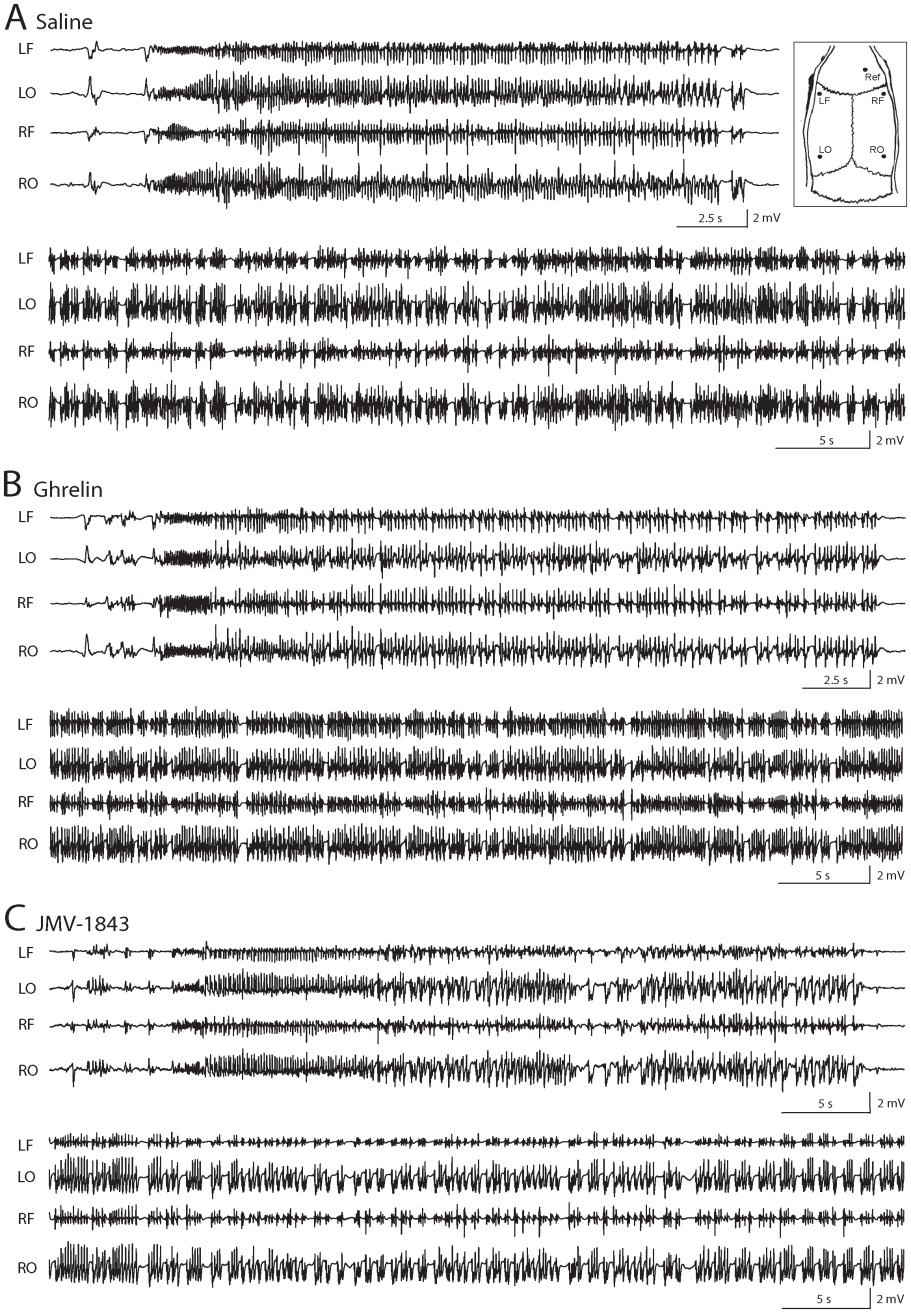
Electrocorticographyc (ECoG) activity in pilocarpine-treated rats. ECoG traces were obtained from 24 pilocarpine-treated rats, from left frontal (LF), left occipital (LO), right frontal (RF) and right occipital (RO) cortices (inset in **A**). Reference electrode (Ref) was in the frontal cortex. Representative ECoG traces of stage 5 seizures (upper traces) and of *status epilepticus* (SE) are recorded from pilocarpine-treated rats receiving saline (**A**), ghrelin (**B**) or JMV-1843 (**C**).

Adult male Sprague-Dawley rats (Harlan, San Pietro al Natisone, UD, Italy), weighing 230–250 g were used. All the experiments were performed in accordance with the European Directive 2010/63/EU and approved by the Italian Ministry of Health (DM 126/2011 - B). All efforts were done to minimize the number of animals and their sufferance. Pilocarpine (380 mg/kg, i.p.; Sigma-Aldrich, Milan, Italy) was used to induce SE. The pilocarpine injection was preceded by methylscopolamine (1 mg/kg, i.p.) to prevent the peripheral effects of cholinergic stimulation [27]. Ghrelin (1.5 mg/kg; n = 14), JMV-1843 (330 µg/kg; n = 13), or saline (n = 13) were injected i.p. 20 min after scopolamine and 10 min before pilocarpine. Doses were chosen according to our previous experiments [25]. All drugs were dissolved in physiological saline. Diazepam (20 mg/kg, i.p.) was injected 10 min after the SE onset to improve survival and to standardize SE duration. This procedure makes convulsive seizures to stop, leaving non-convulsive seizures unaltered for hours [31]. Behavioral (Table 1) and electrographic (Table 2) seizures were carefully evaluated during the experiment and, subsequently, in video electrocorticography (ECoG) recordings. In non-implanted animals, we considered SE as the stage in which rats did not recover normal behavior (i.e., exploration, grooming or motor reaction to stimuli) between one seizure and the other and, according to this criterion, rats were treated with diazepam approximately 25–38 min after the pilocarpine injection. In implanted rats, we considered SE as the stage where epileptiform electrographic activity was virtually continuous (interruption of high amplitude activity was never longer than 5 s; see Figure 1 bottom traces in each panel), a condition reached approximately 16–18 min after pilocarpine injection (Table 2). Non-epileptic rats (n = 8) received methylscopolamine followed, 30 min later, by saline instead of pilocarpine, but not diazepam, and were used as normal control group used for immunohistochemistry. All rats treated with pilocarpine developed SE and were used for immunohistochemistry (n = 16) or for video ECoG (n = 24).

**Table 1:** Latencies to pilocarpine-induced seizures and *status epilepticus* (SE) as assessed by behavioral observation.

		Latency to first behavioral seizure*	Latency to Stage 4–5 behavioral seizure*	Latency to behavioral SE*	Latency to behavioral SE#	Seizures before SE	
						Stage 1–3	Stage 4–5
Saline	n = 8	2.50±0.57	10.38±1.96	18.25±2.84	7.88±1.23	3.75±1.08	4.75±0.77
Ghrelin	n = 8	2.50±0.19	10.75±2.23	16.25±2.78	5.50±1.02	2.63±0.50	3.25±0.49
JMV-1843	n = 8	2.50±0.50	12.38±1.61	18.50±3.05	6.13±1.56	4.75±0.65	3.63±0.50

Behavioral seizures were evaluated according to a modified Racine's scale [31]. Latencies (min) to non-convulsive (stages 1–3) or convulsive (stages 4–5) seizures of Racine's scale were not different among the various treatment groups (*P*>0.05; one-way ANOVA). The delay to SE onset after the first Racine's stage 4–5 seizure was also similar in the different groups of pilocarpine-treated rats. Note also that all rats, independently of drug treatment, experienced non-convulsive followed by convulsive seizures, and then SE.

* t_0_ = pilocarpine injection.

# t_0_ = first behavioral stage 4–5 seizure.

**Table 2:** Latencies to pilocarpine effects and quantification of electrographic (ECoG) seizures preceding *status epilepticus* (SE).

		Latency to first ECoG seizures*	Latency to ECoG Stage 4–5 seizures*	Latency to ECoG SE*	Latency to ECoG SE#	ECoG seizures before SE	
						Stage 1–3	Stage 4–5
Saline	n = 8	8.00±2.07	8.00±2.07	17.38±2.80	9.38±1.16	0.13±0.13	5.75±0.70
Ghrelin	n = 8	8.06±2.18	10.56±2.53	16.63±2.77	6.06±0.85	1.63±0.56	4.00±0.96
JMV-1843	n = 8	8.56±1.99	10.69±1.80	18.00±2.92	7.19±1.41	1.38±0.60	4.13±0.99

Time intervals (min) to the onset of ECoG seizures and SE after the injection of pilocarpine are shown. The delay to SE onset after the first ECoG Racine's stage 4–5 seizure is also illustrated. JMV-1843 and ghrelin did not affect latencies to seizures or to SE onset compared to saline group (*P*>0.05; one-way ANOVA). JMV-1843 and ghrelin did not change the appearance of non-convulsive (stage 1–3) or convulsive seizures (stage 4–5) that preceded SE (*P*>0.05; one-way ANOVA). All rats pretreated with saline, ghrelin and JMV-1843 showed electrographic activity during behavioral stage 4–5 seizures and SE, while electrographic activity was often not detected by superficial electrodes during behavioral stage 1–3 seizures, most likely because activity was limited to deep structures.

* t_0_ = pilocarpine injection.

# t_0_ = first ECoG stage 4–5 seizure.

### ECoG

In vivo video ECoG was performed in a subset of rats (saline, n = 8; ghrelin, n = 8; JMV-1843, n = 8) during pilocarpine-induced SE. Electrodes were implanted as previously described [31]. Briefly, anesthesia was induced by volatile isoflurane (4% induction, 1–2% maintenance), guiding holes were drilled and epidural electrodes (stainless steel Ø = 1 mm; PlasticsOne, Roanoke, VA, USA) were implanted in frontal (bregma 0 mm, 3.5 mm lateral from midline) and occipital cortices (bregma −6.5 mm, 3.5 mm lateral from midline) of both hemispheres (Figure 1A inset). One electrode was implanted in the frontal bone and used as reference (bregma +2.5 mm, 1.5 mm lateral on the right). Electrodes were connected by steel wire to terminal gold pins (Bilaney Consultant GmbH, Düsseldorf, Germany) inserted in a plastic pedestal (PlasticsOne) cemented on the rat head. Electrical brain activity was filtered (0.3 Hz high-pass, 500 Hz low-pass), acquired at 1 kHz per channel, and stored on personal computer as the mathematical subtraction of traces of recording electrodes minus traces of reference electrode using a PowerLab/30 series amplifier (ADInstruments; Dunedin, Otago, New Zealand). ECoG traces were offline digitally filtered (band-pass: high 50 Hz, low 1 Hz) and analyzed by hand scroll using LabChart 7 software (ADInstruments). Videos were digitally captured by a camera connected to the computer and synchronized to the ECoG traces by LabChart 7 internal trigger. Three to 14 days after electrode implantation, rats received the sequence of treatments described above. Rats were recorded for 20 min in methylscopolamine, for 10 min after drug treatment (ghrelin, JMV-1843 or saline), for 10 min after pilocarpine-induced SE, and for 2 h after diazepam administration. Between two consecutive seizures trace returned to baseline for at least 5 s; if interruption of epileptiform activity was less than 5 s, two consecutive electrical activities were considered part of the same seizure. Rats implanted and recorded for ECoG were not used for immunohistochemistry.

### Immunohistochemistry

Pilocarpine-treated rats (n = 5 saline, n = 6 ghrelin, n = 5 JMV-1843) and non-epileptic control rats (n = 8) were anesthetized with chloral hydrate (450 mg/kg, i.p.) 4 days after the injections and perfused through the ascending aorta, using 100 ml saline followed by 100 ml 4% paraformaldehyde and picric acid (0.3%) dissolved in 0.1 M phosphate buffer (pH 6.9). Brains were kept overnight in the same fixative at 4 °C and cryoprotected by immersion in 15% and 30% sucrose-phosphate buffer solutions. They were then frozen and cut horizontally with a freezing microtome in serial 50 µm-thick sections from bregma levels −9.1 mm to −5.1 mm [36]. After several washes, sections were blocked in phosphate buffered saline (PBS, pH 7.4) containing 2% goat serum and were respectively incubated with antibodies able to reveal astrocytic [37], neuronal [38] and vessel wall lesions [39]; in particular, we used three monoclonal antibodies: anti-GFAP (1∶1000, Sigma-Aldrich); anti-neuron-specific nuclear protein (NeuN, 1∶200; Chemicon, Temecula, CA, USA); anti-laminin (1∶2000, Sigma-Aldrich). In addition, we also evaluated the possible induction of ET-1 immunoreactivity by using a polyclonal antibody (1∶5000, Peninsula Laboratories Inc., San Carlos, CA, USA) [33]. Immunostaining was revealed using the avidin–biotin complex technique and diaminobenzidine as chromogen [30].

### Histochemistry

We stained degenerating neurons in adjacent sections to those used for immunohistochemistry, using Fluoro-Jade B (Histo-Chem Inc, Jefferson, AR, USA) as fluorochrome. Brain sections (6 for each animal, cut horizontally with a freezing microtome in serial 50 µm-thick sections from bregma levels −9.1 mm to −5.1 mm) were washed in PBS, mounted on gelatin-coated slides and dried overnight (37 °C). Sections were progressively hydrated by immersion in decreasing ethanol solutions (3 min in absolute ethanol and 1 min in 70% ethanol) and treated with 0.06% potassium permanganate for 15 min. After a 1 min wash in distilled water, sections were stained in a 0.001% Fluoro-Jade B solution containing 0.1% acetic acid. Sections were then washed in water, dried on a pre-warmed hotplate at 37 °C, dipped in xylene and covered with mounting medium (Eukitt, EMS Inc, Hatfield, PA, USA) [40]. Images were acquired with a Zeiss Axioskop 40 fluorescence microscope equipped with an AxioCamHRc digital camera. Fluoro-Jade B positive neurons were counted in the hilus of dentate gyrus (hereafter referred as hilus) and medial entorhinal cortex layer III, and expressed in terms of the sampled area (cells/mm^2^).

### Image analysis

Serial sections were analyzed by the KS300 image analysis software (Carl Zeiss Vision, GmbH-München, Germany) as previously described [30], [41]. Briefly, fields (1.18 mm^2^) of GFAP and laminin immunoreactivity were acquired in CA3 by an Axioskop microscope (Zeiss, Munich, Germany) using a Sony CCD-IRIS BW video camera. GFAP lesions, expressed in mm^2^ for each animal, were delimited as previously illustrated [31] and measured in 6 serial sections separated each other by 0.3 mm, for each hippocampus. After selecting laminin-specific immunostaining [31], immunoreactivity was measured as field area (FA) values, corresponding to the sum of areas with the specific profiles obtained after discrimination from the background staining and expressed in mm^2^. A similar procedure was used for the ET-1 immunostaining in CA3. Neuronal cell profiles were obtained as previously illustrated [42]: after identification by NeuN antibodies, NeuN-positive cells were measured in 0.38 mm^2^ fields within the hilus and layer III of the medial entorhinal cortex of each serial section and expressed as cell densities (n/mm^2^) by considering a minimum cut-off value of 7 µm. In the CA1 region of the hippocampus close to the subiculum (equivalent to the Sommer's sector in the human hippocampus of patients exposed to SE and affected by temporal lobe epilepsy [43]–[44]) and in the CA3b sector, we sampled a smaller area by doubling magnification, in order to clearly distinguish the densely packed NeuN-positive neuronal nuclei from each other. Background values for laminin or NeuN were respectively obtained in hippocampal areas devoid of specific immunostaining. Up to 12 different hippocampal fields/rat were analyzed and values were averaged and used for statistical purposes. Same procedure was used for sections stained by Fluoro-Jade B.

### Statistical analysis

Statistical comparisons were performed by one-way analysis of variance (ANOVA) and followed by the Fisher's least significant difference (LSD) test (SigmaPlot 11, Systat Software, San Josè, CA, USA). Values are presented as mean±SEM and a *P*<0.05 was selected as threshold for significant differences between the means.

## Results

### Induction of SE

In all rats, pilocarpine successfully induced seizures and SE assessed by the experimenter (Table 1) and video ECoG (Table 2). Responses to pilocarpine in rats treated with ghrelin or JMV-1843 were not different from those of saline-treated rats. Behavioral non-convulsive seizures (Table 1) preceded seizures identified by video ECoG (Table 2). Discrepancies between the two analyses were probably due to seizures originating from deep structures that did not propagate to epidural superficial electrodes. Non-convulsive seizures, corresponding to stage 1–3 of the modified Racine's scale [31], were observed in all rats, but these seizures were detected in the ECoG recordings of 12.5%, 75% and 62.5% of rats receiving, respectively, saline, ghrelin and JMV-1843 before pilocarpine injection (Table 2). ECoG analysis revealed that pilocarpine induced a set of approximately 5 convulsive seizures, corresponding to stage 4–5 seizures of the modified Racine's scale [31], in all groups (Table 2; illustrated by ECoG recordings in Figure 1, top traces in each panel). The SE started 8–11 min after first convulsive seizure (Figure 1, bottom traces in each panel; Table 2). Statistical analysis did not reveal significant differences for stage 1–3 or 4–5 seizures.

### GHS-R_1a_ agonists reduce the astrocytic lesion in CA3

**Figure 2:**
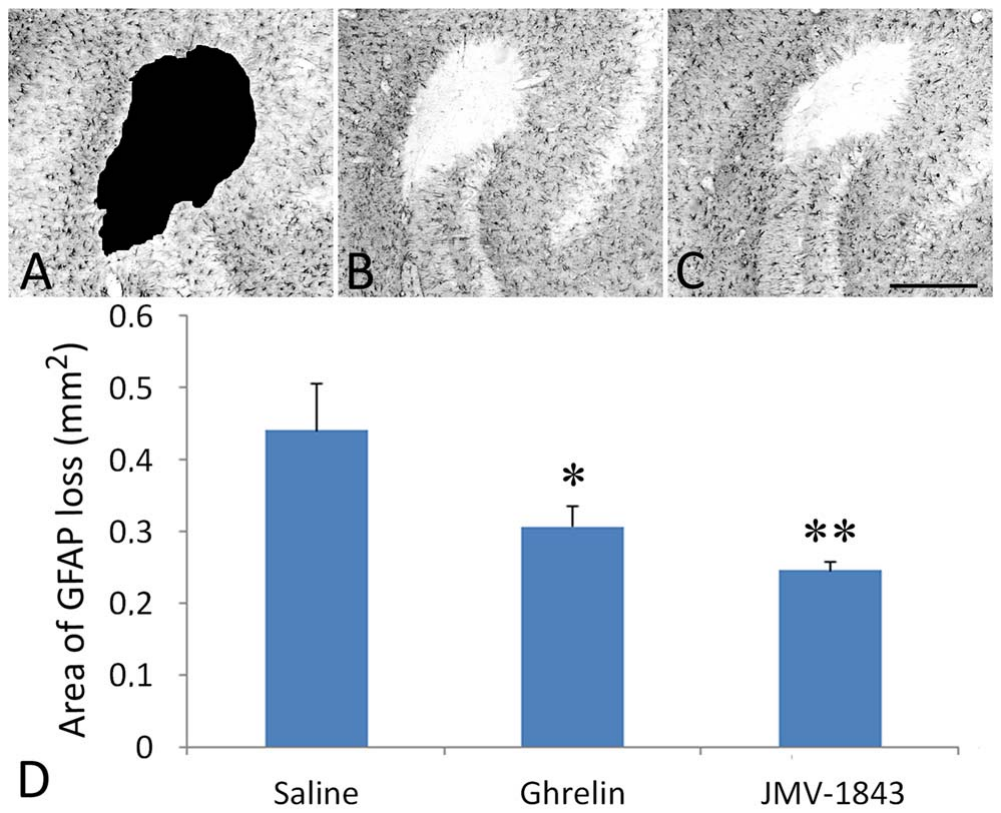
Photomicrographs illustrating the glial lesion appearing in the CA3 stratum lacunosum-moleculare after *status epilepticus* (SE), in pilocarpine-treated rats. The lesion was investigated using an antibody against the glial fibrillary acidic protein (GFAP), which specifically stains astrocytes. In a pilocarpine-treated rat of the saline-treated group, sacrificed 4 days after SE, the GFAP immunostaining is completely abolished in the core of the lesion, which is surrounded by strongly immunopositive reactive astrocytes (A-C). The lesion core was manually demarcated and its area measured, as indicated by the blackish area in A. Pretreatment with the GH secretagogue ghrelin (B) and JMV-1843 (C) resulted in less marked lesions, as shown in D. * = *P*<0.05, ** = *P*<0.01 vs the saline group, Fisher's LSD test. Scale bar, 300 µm.

Following SE, a focal lesion, characterized by the complete disappearance of GFAP immunoreactivity and surrounded by intensely-labelled reactive astrocytes, was found in the CA3 stratum lacunosum-moleculare of saline-treated rats (Figure 2A), confirming our previously described findings [30]–[31]. Although this lesion was not prevented by any of the tested GHS-R_1a_ agonists, it was significantly reduced in both ghrelin and JMV-1843 treatment groups (Figure 2B,C). In particular, the mean area of GFAP loss, represented as% compared to saline-treated rats, was 70% in the ghrelin group (*P*<0.05 vs saline) and 57% in the JMV-1843 group (*P*<0.01 vs saline) (Figure 2D).

### JMV-1843 prevents increase in laminin and ET-1 levels occurring after SE

**Figure 3:**
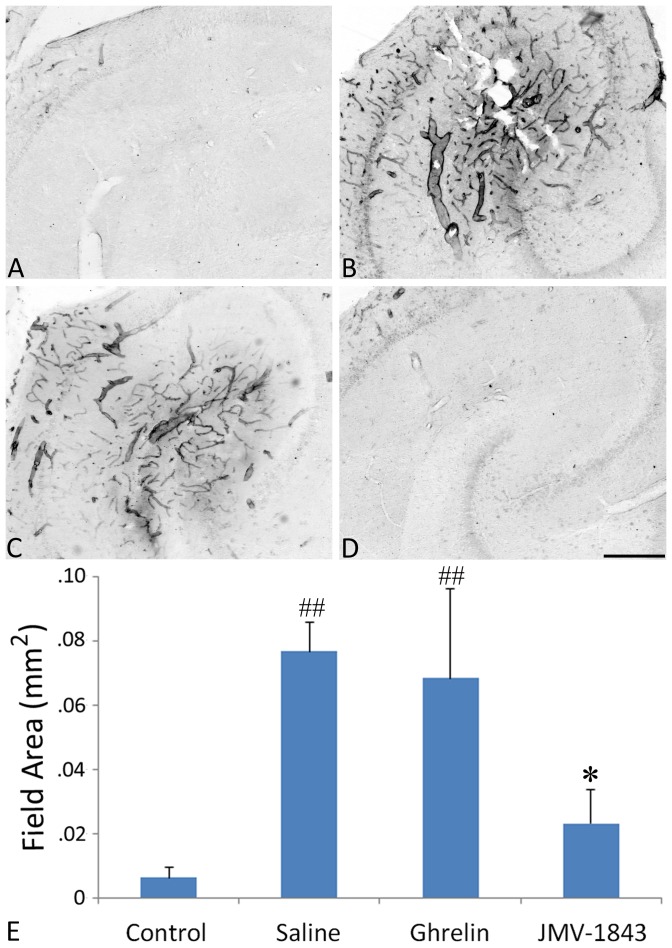
Photomicrographs illustrating the vascular lesion appearing in the CA3 stratum lacunosum-moleculare after *status epilepticus* (SE), in pilocarpine-treated rats. The lesion was investigated using an antibody to laminin, which identifies the basal lamina in blood vessels in control tissue (A). Laminin immunoreactivity is markedly upregulated in the damaged area of a pilocarpine-treated rat of the saline-treated group (B) sacrificed 4 days after SE. Pretreatment with the GH secretagogue ghrelin (C) did not affect the increase of laminin immunoreactivity. Notably, pretreatment with JMV-1843 (D) prevented the changes observed in the other treatment groups, which are quantified in E. ^##^ = *P*<0.01 vs the control non-epileptic group,* = *P*<0.05 vs the saline pilocarpine-treated group, Fisher's LSD test. Scale bar, 300 µm.

We previously demonstrated that the loss of GFAP immunostaining in the CA3 region of pilocarpine-treated rats is highly associated with an increase in laminin immunoreactivity in the basal lamina of blood vessels [31]. We thus evaluated whether treatment with GHS-R_1a_ agonists would also affect the laminin immunoreactivity in the hippocampus (Figure 3). In the saline group of pilocarpine-treated rats (Figure 3B), laminin levels were 12-fold higher than those measured in normal control rats (*P*<0.01; Figure 3A,E). Although laminin levels were also increased in rats treated with ghrelin (*P*<0.01 vs non-epileptic controls; Figure 3C), this change was completely prevented by JMV-1843 (Figure 3D). In fact, laminin levels in JMV-1843-treated rats were not significantly different from those found in control non-epileptic rats (Figure 3A) and lower than in saline-treated pilocarpine rats (*P*<0.05; Figure 3B,E).

**Figure 4:**
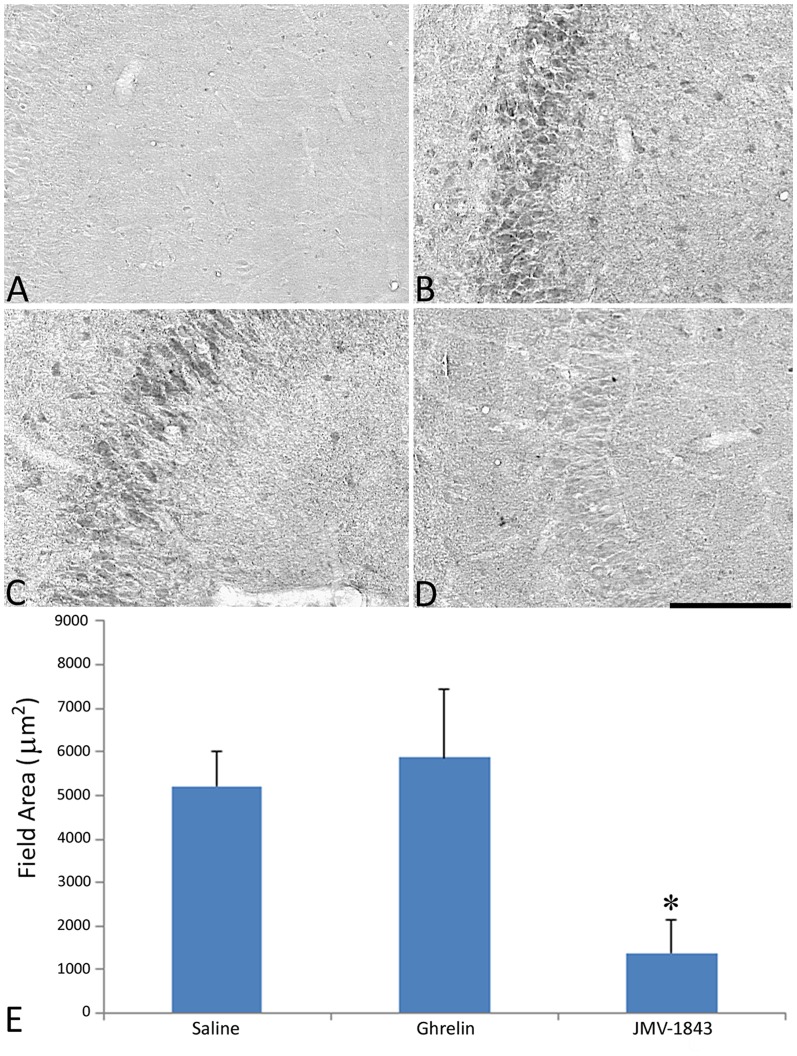
Changes in endothelin-1 (ET-1) immunoreactivity in the CA3 region of pilocarpine-treated rats after *status epilepticus* (SE). ET-1 is barely detectable in control non-epileptic rats (A). Immunoreactivity for ET-1 was strongly induced by the pilocarpine injection (B, illustrating a saline-treated rat), and ghrelin did not affect the remarkable induction of ET-1 (C). On the contrary, JMV-1843 counteracted the induction of ET-1 in the CA3 pyramidal cell layer (D, E). * = *P*<0.05 vs both saline and ghrelin groups, Fisher's LSD test. Scale bar, 150 µm.

In view of the remarkable upregulation of laminin immunoreactivity found after intracerebral injection of ET-1 [31] and of the induction of this vasoactive neuropeptide after pilocarpine-induced SE [33], we analyzed the effects of pilocarpine and the GHS-R_1a_ agonists on ET-1 immunoreactivity in CA3. ET-1 immunopositivity was barely detectable in control non-epileptic rats (Figure 4A). However, a prominent upregulation was observed in consequence of pilocarpine-induced SE (Figure 4B). This change was particularly evident in CA3 pyramidal neurons. Ghrelin administration did not modify the increase of ET-1 immunoreactivity (Figure 4C). On the contrary, JMV-1843 significantly counteracted the increase of ET-1 immunoreactivity in pilocarpine-treated rats (*P*<0.05 vs saline and ghrelin groups; Figure 4D,E).

### JMV-1843 protects neurons against cell death in the hilus and, to a lesser extent, in the entorhinal cortex

**Figure 5:**
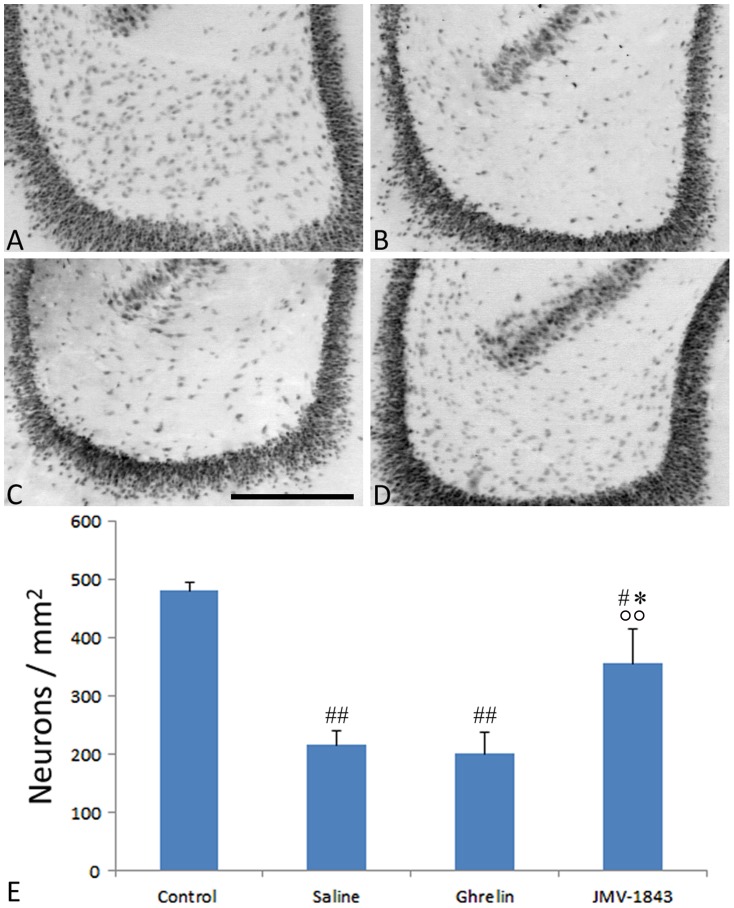
Photomicrographs illustrating neuronal cell loss in the hilus of dentate gyrus after *status epilepticus* (SE), in pilocarpine-treated rats. Neurons were identified by the anti-neuron-specific nuclear protein (NeuN) antibody, as shown in the control staining (A). NeuN-immunopositive cells were markedly decreased in pilocarpine-treated rat of the saline- (B) and ghrelin-treated groups (C), sacrificed 4 days after SE. This phenomenon was significantly counteracted by JMV-1843 administration (D). Neuronal cell counts are shown in E. ^#^ = *P*<0.05, ^##^ = *P*<0.01 vs the control non-epileptic group, * = *P*<0.05 vs the saline group, °° = *P*<0.01 vs the ghrelin group, Fisher's LSD test. Scale bar, 300 µm.

To evaluate possible protective effects of GHS-R_1a_ agonists on neurons, we counted NeuN-positive cells in the hippocampal formation. Neuronal cell density in the hilus (Figure 5A), a region in which neuronal cell loss is consistently reported in pilocarpine-treated rats [27], was reduced to approximately 45% of control levels (*P*<0.01) in the saline group of pilocarpine-treated rats (Figure 5B). Hilar neuronal densities were also decreased in ghrelin-treated rats (*P*<0.01 *vs* control levels; Figure 5C). In contrast, JMV-1843 had a protective effect. Indeed, JMV-1843-treated rats presented neuronal cell density values corresponding to approximately 74% of those found in control non-epileptic rats (*P*<0.05, Figure 5D). Accordingly, neuronal cell density was significantly higher in JMV-1843 compared with saline- (*P*<0.05, Figure 5E) and ghrelin-treated (*P*<0.01) rats.

**Figure 6:**
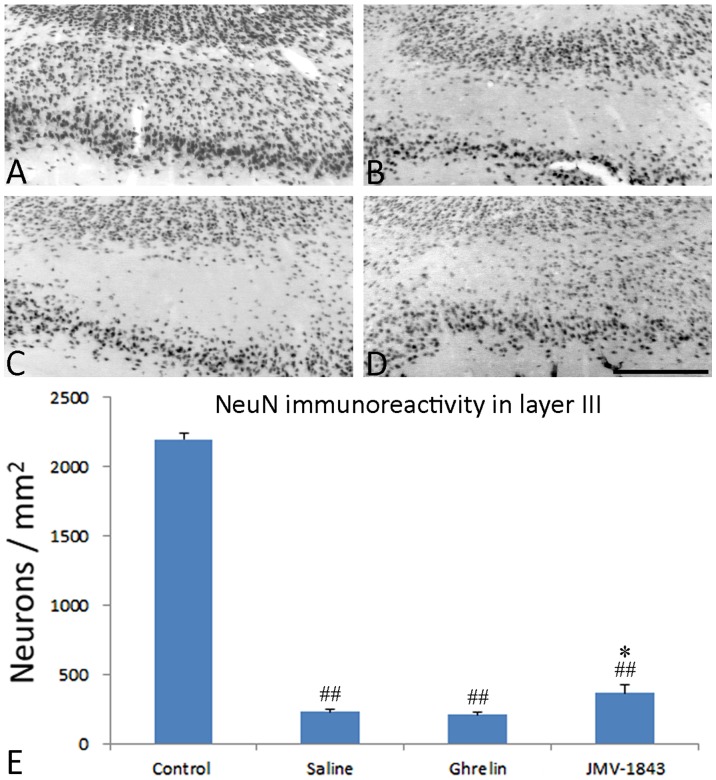
Photomicrographs illustrating neuronal cell loss in the medial entorhinal cortex layer III after *status epilepticus* (SE), in pilocarpine-treated rats. Neurons were identified by the anti-neuron-specific nuclear protein (NeuN) antibody, as shown in the control staining (A). NeuN-immunopositive cells were markedly decreased in pilocarpine-treated rat of the saline-treated group, sacrificed 4 days after SE (B). Similar findings were found in rats treated with ghrelin before receiving pilocarpine (C). This phenomenon was significantly counteracted by JMV-1843 administration (D). Neuronal cell counts are shown in E. ^##^ = *P*<0.01 vs the control non-epileptic group, * = *P*<0.05 vs both the saline and ghrelin groups, Fisher's LSD test. Scale bar, 300 µm.

We then evaluated neuronal cell densities in the medial entorhinal cortex layer III (Figure 6), another region of the hippocampal formation particularly vulnerable to pilocarpine-induced damage [27], [32]. Neurons in layer III were decreased to approximately 10% of control levels (*P*<0.01) in the saline group of pilocarpine-treated rats (cf. Figure 6A and 6B). Similar decreased values were found in ghrelin-treated rats (*P*<0.01 *vs* control levels; Figure 6C). Again, JMV-1843 had protective effect (Figure 6D), although much smaller than the one observed in the hilus. JMV-1843-treated rats were also characterized by decreased neuronal cell counts, corresponding to approximately 20% of values found in control non-epileptic rats (*P*<0.01). However, neuronal cell density was significantly (*P*<0.05) higher (+60−70%) in the JMV-1843 group compared with saline- or ghrelin-treated rats (Figure 6E).

**Figure 7:**
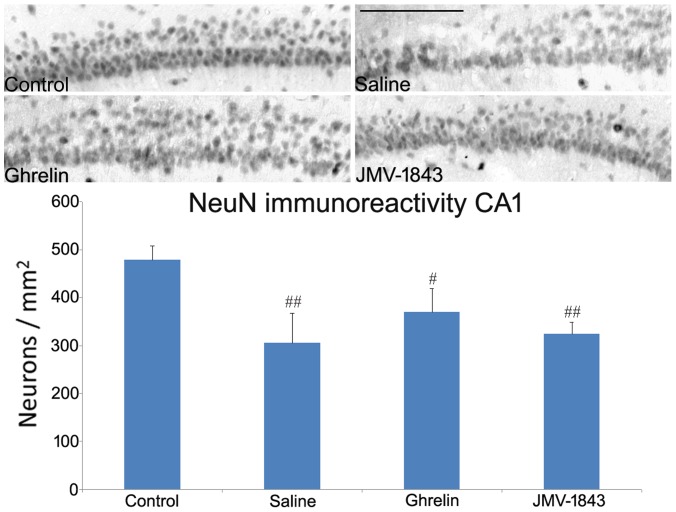
Photomicrographs illustrating neuronal cell loss in the CA1 hippocampal region after *status epilepticus* (SE), in pilocarpine-treated rats. Neurons were identified by the anti-neuron-specific nuclear protein (NeuN) antibody, as shown in the control staining. NeuN-immunopositive cells were markedly decreased in pilocarpine-treated rat of the saline-treated group, sacrificed 4 days after SE. This phenomenon was not attenuated by treatment with GH secretagogues. ^#^ = *P*<0.05, ^##^ = *P*<0.01 vs the control non-epileptic group, Fisher's LSD test. Scale bar, 150 µm.

**Figure 8:**
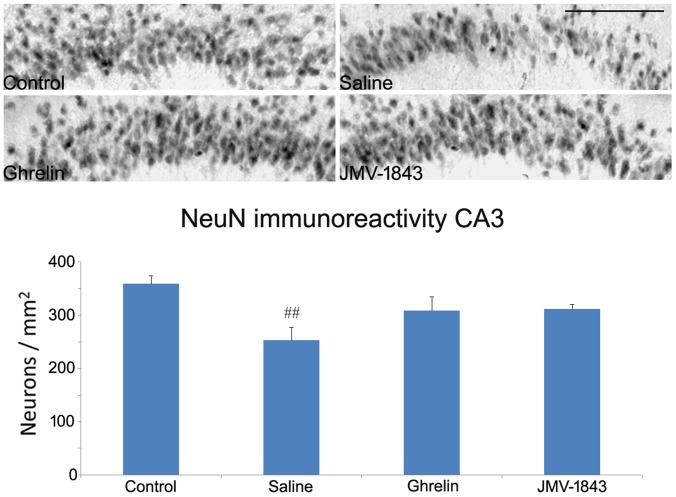
Photomicrographs illustrating neuronal cell loss in the CA3 hippocampal region after *status epilepticus* (SE), in pilocarpine-treated rats. Neurons were identified by the anti-neuron-specific nuclear protein (NeuN) antibody, as shown in the control staining. NeuN-immunopositive cells were markedly decreased in pilocarpine-treated rats of the saline group, sacrificed 4 days after SE. This phenomenon was attenuated by treatment with GH secretagogues.^ ##^ = *P*<0.01 vs the control non-epileptic group, Fisher's LSD test. Scale bar, 150 µm.

A different scenario emerged from the analysis of neuronal cell densities in the hippocampus proper. The highly packed distribution of neurons in the CA1 and CA3 regions did not allow a similar analysis to the one performed in the hilus of dentate gyrus and medial entorhinal cortex layer III. Thus, we sampled at higher magnification the CA1 sector proximal to the subiculum (Figure 7) and the CA3b sector (Figure 8), which represent for each respective region the most sensitive areas to damage in rat exposed to SE [27], [31]. In CA1, we found that NeuN-positive cells were decreased in the saline group to approximately 65% of control levels (*P*<0.01). A similar decrease was observed in GHS-R_1a_ agonist-treated rats: NeuN-positive cells were decreased, respectively, to 77% in ghrelin (*P*<0.05 vs controls) and to 67% of control levels in JMV-1843 (*P*<0.01) groups. Contrary to what we observed in the hilus and entorhinal cortex, JMV-1843 did not have any protective effect in the CA1.

In sector CA3b, neuronal cell density decreased to 70% of control values (*P*<0.05) in saline-treated rats. In rats treated with GHS-R_1a_ agonists, neuronal cell densities were also decreased but did not reach significant statistical difference (Figure 8), suggesting that GHS-R_1a_ agonists might also protect CA3b neurons against cell death.

**Figure 9:**
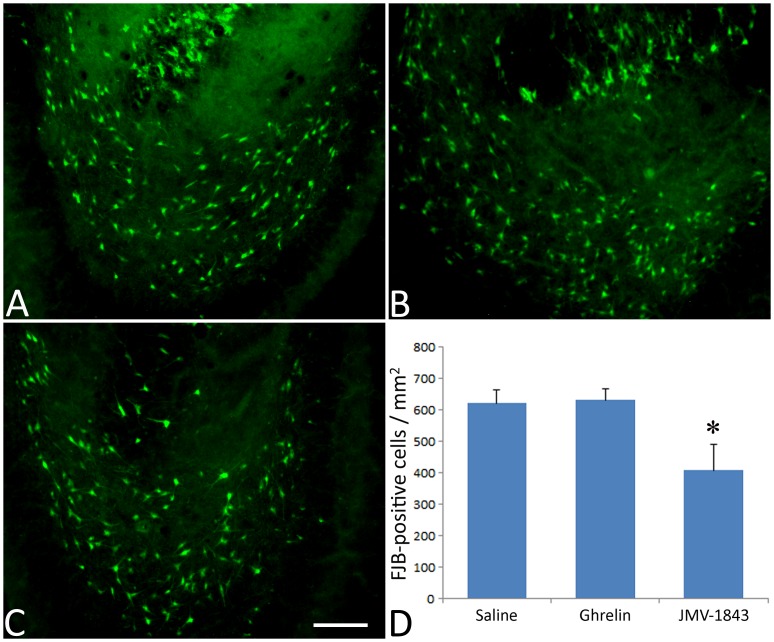
Photomicrographs illustrating the lesion appearing in the hilus of dentate gyrus after *status epilepticus* (SE), in pilocarpine-treated rats. The lesion was investigated using the Fluoro-Jade B staining in saline (A), ghrelin (B) and JMV-1843 (C) groups. JMV-1843 administration decreased the number of Fluoro-Jade B-positive cells (D).* = *P*<0.05 vs both saline and ghrelin groups, Fisher's LSD test. Scale bar, 100 µm.

**Figure 10:**
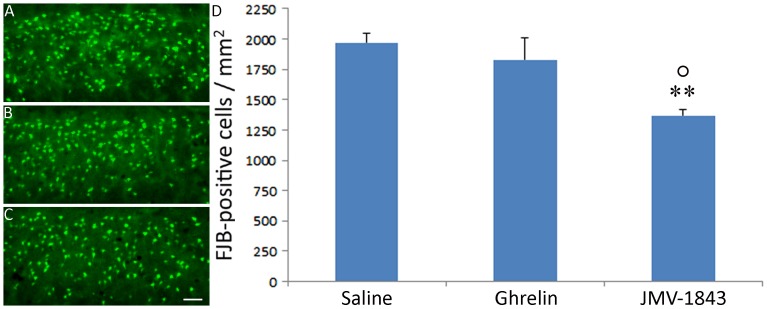
Photomicrographs illustrating the lesion appearing in the medial entorhinal cortex layer III, after *status epilepticus* (SE), in pilocarpine-treated rats. The lesion was investigated using the Fluoro-Jade B staining in saline (A), ghrelin (B) and JMV-1843 (C) groups. Note that JMV-1843 administration decreased the number of Fluoro-Jade B-positive cells (D). ** = *P*<0.01 vs the saline group, ° = *P*<0.05 vs the ghrelin group, Fisher's LSD test. Scale bar, 100 µm.

NeuN has been used often as a specific marker of neuronal cell loss and it provided coherent results both in pilocarpine [45] and in kainate models [46]. However, caution must be used when assuming changes in NeuN immunoreactivity as a direct index of neuronal cell loss [38]. Thus, we evaluated damaged cells by Fluoro-Jade B, a consistent marker of cell death [40]. No stained cells were observed in control animals (data not shown). In contrast, positively stained cells were always observed in pilocarpine rats treated with either saline or GHS-R_1a_ agonists. In general, findings obtained with the Fluoro-Jade B staining confirmed results obtained with NeuN immunoreactivity. More precisely, in the hilus (Figure 9), JMV-1843-treated animals displayed approximately 35% less fluorescent cells compared to saline- or ghrelin-treated rats (*P*<0.05). A similar scenario was found also in the medial entorhinal cortex, layer III (Figure 10), where the level of fluorescent cells in the JMV-1843 group was significantly lower (approximately −30%) in comparison with that of the saline (*P*<0.01) and ghrelin (*P*<0.05) groups.

## Discussion

Ghrelin is an endogenous ligand of GHS-R_1a_ found to exert protective actions against neuronal cell death in various experimental models, including hypoxia and glucose deprivation [19], cerebral ischemia [20]–[22], and SE [23]–[24]. In these models, ghrelin inhibited apoptosis by decreasing induction of caspase 3 activity [23]–[24]. This antiapoptotic property was also demonstrated in cells other than neurons, such as endothelial cells [16]. Furthermore, ghrelin is capable to preserve tissue vascularization, as demonstrated in the retina, where ghrelin prevented obliteration of capillaries due to hyperoxia [47]. Moreover, hexarelin and other GH secretagogues are endowed with positive cardiovascular effects [15], [18]. For these reasons, we investigated the possible beneficial effects of ghrelin and the GHS-R_1a_ agonist JMV-1843 in the pilocarpine model of SE, in which both apoptosis [48]–[50] and vascular damage [30]–[31], [51]–[53] coexist.

Initially, we evaluated the effects of ghrelin and JMV-1843 on the vascular lesion that occurs in the CA3 hippocampal region after pilocarpine treatment [30]–[31]. This lesion is characterized by a double pathological hallmark, consisting in the complete disappearance of GFAP immunostaining, as observed in the core lesion of an infarct, and the upregulation of laminin immunoreactivity in blood vessels. This phenomenon is probably related to disconnection of the glia *limitans* from the basal lamina of blood vessels, leading to disruption of the blood-brain barrier [54]–[55] and is reproduced by intracerebral injection of ET-1 [31]. Interestingly, we found that JMV-1843 was able to prevent the changes in laminin immunoreactivity.

We also tried to define a possible mechanism for the observed protective effects of GHS-R_1a_ agonists on blood vessels in CA3. We have previously shown that an intracerebral injection of ET-1 is able to reproduce the changes of laminin and GFAP immunoreactivities observed in CA3 after SE [31]. Thus, we evaluated whether ET-1 could be induced in pilocarpine-treated rats, as suggested by Jo et al. [33]. We found that ET-1 is highly expressed in the CA3 of pilocarpine-treated rats and JMV-1843 is able to markedly prevent the upregulation of ET-1. This finding is consistent with data obtained in other tissues different from the brain. Indeed, exogenous administration of ghrelin for 2 weeks was found to prevent the upregulation of ET-1 occurring after chronic pulmonary hypertension in rats [56]. Consistently, ghrelin administered for 4 weeks to rats that experienced a myocardial infarction attenuated the changes in ET-1 mRNA observed in saline-treated rats [57]. Ghrelin was also found to ameliorate vascular perfusion during sepsis by downregulating ET-1 [35]. Overall, these data support the hypothesis that the potent modulatory properties of ghrelin on arterial blood vessels are mediated through the antagonism of ET-1 activity [34]. This mechanism might contribute to preserve the integrity of basal lamina in blood vessels of the CA3 region of rats treated with JMV-1843.

We evaluated neuronal integrity by counting neurons stained by anti-NeuN antibodies. Although NeuN is universally used as a marker for the evaluation of neuronal cell damage in different brain lesion models [38], [45]–[46], NeuN immunopositivity has been reported also in dying cells [58]. Thus, we also identified damaged cells using the Fluoro-Jade B fluorochrome, confirming the findings obtained with the NeuN immunostaining. We observed a remarkable preservation of NeuN-immunopositive cell densities in regions in which damage was more pronounced, such as the hilus and the layer III of medial entorhinal cortex, only in rats treated with JMV-1843. In contrast, in regions with less marked lesions, such as the hippocampal areas CA1 and CA3, both ghrelin and JMV-1843 induced similar effects. Previous studies demonstrated significant neuroprotective effects in the CA1 and CA3 regions of rats receiving ghrelin 30 min before pilocarpine [24]. The discrepancies between our results and findings by Xu et al. [24] could be explained partly by the different duration of SE (10 min in our experiments vs 30 min in Xu et al. [24]). In any case, ghrelin was beneficial in both the pilocarpine [24] and kainate models of SE [23], an effect confirmed by our present findings, at least in the CA3 region. In addition, we found an even more pronounced neuroprotective effect by using a more efficient agonist of GHS-R_1a_, namely JMV-1843. JMV-1843 is known to possess a better pharmacokinetics than ghrelin [11]–[13]. In fact, single doses of JMV-1843 were shown to induce higher peaks of GH, lasting up to 120 min [59]. These differences could account for the better neuroprotective performance that we observed in the hilus and entorhinal cortex in JMV-1843-treated rats.

In conclusion, we show that pretreatment with GHS-R_1a_ agonists results in beneficial effects in pilocarpine-treated rats exposed to SE. The neuroprotection afforded by ghrelin and JMV-1843 was completely independent of anticonvulsant effects, as shown by the ECoG recordings. JMV-1843, which is a peptidomimetic orally active ligand of the GHS-R_1a_, was more effective than ghrelin. Interestingly, this difference in protection was associated with repression of ET-1 synthesis in perilesional cells, suggesting that this phenomenon is involved in neuroprotection during prolonged seizure exposure.
